# Entropy spikes as a signature of Lifshitz transitions in the Dirac materials

**DOI:** 10.1038/s41598-017-10643-0

**Published:** 2017-08-31

**Authors:** V. Yu. Tsaran, A. V. Kavokin, S. G. Sharapov, A. A. Varlamov, V. P. Gusynin

**Affiliations:** 1 Institut für Kernphysik, Johannes Gutenberg Universität, D-55128 Mainz, Germany; 2 CNR-SPIN, University “Tor Vergata”, Viale del Politecnico 1, I-00133 Rome, Italy; 3 Physics and Astronomy School, University of Southampton, Highfield, Southampton SO171BJ UK; 4 Bogolyubov Institute for Theoretical Physics, National Academy of Science of Ukraine, 14-b Metrolohichna Street, Kiev, 03680 Ukraine

## Abstract

We demonstrate theoretically that the characteristic feature of a 2D system undergoing *N* consequent Lifshitz topological transitions is the occurrence of spikes of entropy per particle *s* of a magnitude ±ln2/(*J* − 1/2) with 2 ≤ *J* ≤ *N* at low temperatures. We derive a general expression for *s* as a function of chemical potential, temperature and gap magnitude for the gapped Dirac materials. Inside the smallest gap, the dependence of *s* on the chemical potential exhibits a dip-and-peak structure in the temperature vicinity of the Dirac point. The spikes of the entropy per particles can be considered as a signature of the Dirac materials. These distinctive characteristics of gapped Dirac materials can be detected in transport experiments where the temperature is modulated in gated structures.

## Introduction

Entropy is an important fundamental property of many-body systems. It governs their thermodynamics, heat transfer, thermoelectric and thermo-magnetic properties. On the other hand, the entropy was always hard to be directly measured experimentally. It has been revealed very recently that the entropy per particle, ∂*S*/∂*n*, where *n* is the electron density, can be experimentally studied^1^. To be more precise, the measured quantity is the temperature derivative of the chemical potential, ∂*μ*/∂*T*, which may be extracted by modulating the temperature of the gated structure with a 2D electron gas playing the role of one of the plates of a capacitor. Both derivatives are equal as a consequence of the Maxwell relation

1 $$s={(\frac{{\rm{\partial }}S}{{\rm{\partial }}n})}_{T}=-{(\frac{{\rm{\partial }}\mu }{{\rm{\partial }}T})}_{n}.$$

In the theoretical paper^2^, quite surprisingly, it has been pointed out that in a quasi-two-dimensional electron gas (2DEG) with parabolic dispersion the entropy per electron, *s*, exhibits quantized peaks at resonances between the chemical potential and size quantization levels. The amplitude of such peaks in the absence of scattering depends only on the subband quantization number and is independent of material parameters, shape of the confining potential, electron effective mass, and temperature.

The quantization of entropy per electron was interpreted in ref. 2 as a signature of the Lifshitz electronic topological transition^3^, which in the 2D case is characterised by a discontinuity in the electronic density of states (DOS). The latter is caused by a change of the topological properties, viz. connectivity of the electronic Fermi surface^4^. Lifshitz transitions widely occur in multi-valley semimetals, doped semiconductor quantum wells, multi-band superconducting systems such as iron-pnictide compounds^5^ and also in 2D Dirac materials, as we discuss below.

In this Report, we analyze theoretically the behavior of the entropy per particle as a function of the chemical potential in a gapped graphene deposited on a substrate and other low-buckled Dirac materials, e.g. silicene and germanene. We show that the entropy per electron in these systems acquires quantized universal values at low temperatures if the chemical potential passes through the edge of consequent gaps. It is a universal property of electronic systems characterised by a step-like behaviour of the density of states. If the chemical potential is resonant to the Dirac point, we find the discontinuity in *s* at very low temperature. At low but finite temperatures this discontinuity transforms into the combination of a very sharp dip at the negative chemical potential followed by a sharp peak at the positive chemical potential. These predictions offer a new tool for the characterisation of novel crystalline structures. In particular, the very characteristic spikes of entropy that must be relatively easy to observe are indicative of the consequent gaps, in particular due to spin-orbit interaction. We believe that the measurements of the entropy per particle (e.g. following the technique of ref. 1) may reveal hidden peculiarities of the band structure of new materials.

## Results

### The link between the discontinuity of the DOS and the quantization of entropy

To start with, let us consider an electronic system characterised by a DOS function *D*(*ε*) that has a discontinuity. In order to describe Dirac materials specifically, we assume that the DOS is a symmetric function, *D*(*ε*) = *D*(−*ε*), although this assumption is not essential. We shall assume that the DOS has 2*N* discontinuities at the points *ε* = ±Δ_*i*_ and it can be presented in the form

2 $$D(\varepsilon )=f(\varepsilon )\sum _{i\mathrm{=1}}^{N}\theta ({\varepsilon }^{2}-{{\rm{\Delta }}}_{i}^{2})\mathrm{.}$$

The function *f*(*ε*) is assumed to be a continuous even function of energy *ε* and it may account for the renormalizations due to electron-electron interactions in the system.

The case of *N* = 1 corresponds to a gapped graphene with the dispersion law $\varepsilon (k)=\pm \sqrt{{\hslash }^{2}{v}_{F}^{2}{k}^{2}+{{\rm{\Delta }}}^{2}}$ and $f(\varepsilon \mathrm{)=2|}\varepsilon |/(\pi {\hslash }^{2}{v}_{F}^{2})$, where we have taken into consideration both the valley and spin degeneracy. Here Δ is the gap, *v*_*F*_ is the Fermi velocity, *k* is the wavevector. The global sublattice asymmetry gap 2Δ ~ 350 K can be introduced in graphene^6–9^ if it is placed on top of a hexagonal boron nitride (G/hBN) and the crystallographic axes of graphene and hBN are aligned.

The case of *N* = 2 corresponds to silicene^10^, germanene^11^ and other low-buckled Dirac materials^12, 13^. The dispersion law in these materials writes ${\varepsilon }_{\eta \sigma }(k)=\pm \sqrt{{\hslash }^{2}{v}_{F}^{2}{k}^{2}+{{\rm{\Delta }}}_{\eta \sigma }^{2}}$, where *η* and *σ* are the valley and spin indices, respectively. Here the valley- and spin-dependent gap, Δ_*ησ*_ = Δ_*z*_ − *ησ*Δ_SO_, where Δ_SO_ is the material dependent spin-orbit gap caused by a strong intrinsic spin-orbit interaction. It has a relatively large value, e.g. Δ_SO_ ≈ 4.2 meV in silicene and Δ_SO_ ≈ 11.8 meV in germanene. The adjustable gap Δ_*z*_ = *E*_*z*_ *d*, where 2*d* is the separation between the two sublattices situated in different planes, can be tuned by applying an electric field *E*_*z*_. The function $f(\varepsilon )=|\varepsilon |/(\pi {\hslash }^{2}{v}_{F}^{2})$ is twice smaller than one for graphene, because the first transition in Eq. (2) with *i* = 1 corresponds to *η* = *σ* = ± with Δ_1_ = |Δ_SO_ − Δ_*z*_| and the second one with *i* = 2 corresponds to *η* = −*σ* = ± with Δ_2_ = |Δ_*z*_ + Δ_SO_|.

Since the DOS is a symmetric function, instead of the total density of electrons it is convenient to operate with the difference between the densities of electrons and holes (see the Methods) given by

3 $$n(T,\mu ,{{\rm{\Delta }}}_{1},{{\rm{\Delta }}}_{2},\ldots ,{{\rm{\Delta }}}_{N})=\frac{1}{4}{\int }_{-{\rm{\infty }}}^{{\rm{\infty }}}d\varepsilon D(\varepsilon )[\tanh \,\frac{\varepsilon +\mu }{2T}-\,\tanh \,\frac{\varepsilon -\mu }{2T}],$$

where we set *k*_*B*_ = 1. Clearly, *n*(*T*, *μ*) is an odd function of *μ* and *n*(*T*, *μ* = 0) = 0. The density *n* in the Dirac materials may be controlled by an applied gate voltage. In what follows we consider the dependence of *s* on the chemical potential.

As it was mentioned above, the entropy per particle is directly related to the temperature derivative of the chemical potential at the fixed density *n* (see Eq. (1)). The latter can be obtained using the thermodynamic identity

4 $${(\frac{{\rm{\partial }}\mu }{{\rm{\partial }}T})}_{n}=-{(\frac{{\rm{\partial }}n}{{\rm{\partial }}T})}_{\mu }{(\frac{{\rm{\partial }}n}{{\rm{\partial }}\mu })}_{T}^{-1}.$$

If the chemical potential is situated between the discontinuity points, Δ_*i*_ < |*μ*| < Δ_*i*+1_, and *T* → 0, one obtains for the first derivative in Eq. (4) (see the Methods)

5 $$\frac{\partial n(T,\mu )}{\partial T}=D^{\prime} (|\mu |)\frac{{\pi }^{2}T}{3}\,{\rm{sign}}(\mu ),\quad {{\rm{\Delta }}}_{i}\mathrm{ > 0.}$$

On the other hand, at the discontinuity points *μ* = ±Δ_*J*_ at *T* → 0, one finds

6 $${\frac{\partial n(T,\mu )}{\partial T}|}_{\mu =\pm {{\rm{\Delta }}}_{J}}=\pm [D({{\rm{\Delta }}}_{J}+\mathrm{0)}-D({{\rm{\Delta }}}_{J}-\mathrm{0)}]{\int }_{0}^{\infty }\frac{x\,dx}{{\cosh }^{2}x}=\pm f({{\rm{\Delta }}}_{J})\mathrm{ln}\,2.$$

One can see that a factor of ln2 originates from the integration of the derivative of the Fermi distribution (or $\frac{1}{2}$tanhz) multiplied by the energy. If *μ* = ±Δ_*J*_ with *J* < *N* and *T* → 0 for the second derivative in Eq. (4), one obtains (see the Methods)

7 $${\frac{\partial n(T,\mu )}{\partial \mu }|}_{\mu =\pm {{\rm{\Delta }}}_{J}}=f({{\rm{\Delta }}}_{J})\sum _{i=1}^{N}\theta ({{\rm{\Delta }}}_{J}^{2}-{{\rm{\Delta }}}_{i}^{2})=f({{\rm{\Delta }}}_{J})(J-\mathrm{1/2),}$$

where the first *J* − 1 *θ* functions give *J* − 1 and the last one gives the 1/2 contribution.

Thus, we arrive to the conclusion that the entropy per particle in Dirac materials is

8 $$s(T\to 0,\mu =\pm {{\rm{\Delta }}}_{J})=\pm \frac{\mathrm{ln}\,2}{J-1/2},\quad J=1,2,\ldots N,$$

while for Δ_*i*_ < |*μ*| < Δ_*i*+1_ it vanishes. One can see that the behaviour of entropy per particle for the gapped Dirac systems as a function of chemical potential is analogous to one found in quasi-2DEG with a parabolic dispersion^2^. This fact allows us to speculate that such universal spikes are related rather to the topological changes of the Fermi surface than to specific form of the spectrum.

### Gapped Dirac materials

In the particular case of a gapped graphene the integral (3) can be done analytically^14^

9 $$n(T,\mu ,{\rm{\Delta }})=\frac{2{T}^{2}}{\pi {\hslash }^{2}{v}_{F}^{2}}[\frac{{\rm{\Delta }}}{T}\,{\rm{l}}{\rm{n}}\,\frac{1+\exp (\frac{\mu -{\rm{\Delta }}}{T})}{1+\exp (-\frac{\mu +{\rm{\Delta }}}{T})}+{{\rm{L}}{\rm{i}}}_{2}(-{e}^{-\frac{\mu +{\rm{\Delta }}}{T}})-{{\rm{L}}{\rm{i}}}_{2}(-{e}^{\frac{\mu -{\rm{\Delta }}}{T}})],$$

where *Li* is the polylogarithm function. The derivatives (∂*n*/∂*T*)_*μ*_ and (∂*n*/∂*μ*)_*T*_ are calculated in the Methods, Eqs (22) and (23).

The density of carriers in silicene can be described with use of the formalism developed above for graphene by formally representing silicence as a superposition of two gapped graphene layers characterised by different gaps: *n*(*T*, *μ*, Δ_1_, Δ_2_) = 1/2[*n*(*T*, *μ*, Δ_1_) + *n*(*T*, *μ*, Δ_2_)].

**Figure 1:**
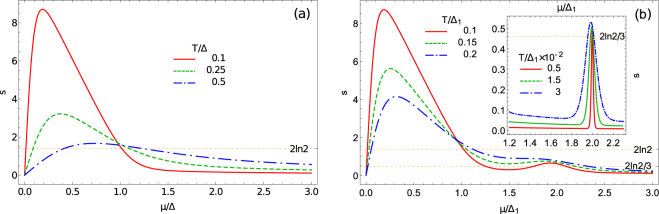
The entropy per electron *s* vs the chemical potential *μ* > 0, *s*(−*μ*) = −*s*(*μ*), for three values of temperature. Left panel: (a): Gapped graphene. The chemical potential *μ* is expressed in the units of Δ; the solid (red) *T*/Δ = 0.1, dashed (green) *T*/Δ = 0.25, dash-dotted (blue) *T*/Δ = 0.5. Right panel: (b): Silicene. *μ* is in the units of a smaller gap Δ_1_, the second gap Δ_2_ = 2Δ_1_; the solid (red) *T*/Δ_1_ = 0.1, dashed (green) *T*/Δ_1_ = 0.15, dash-dotted (blue) *T*/Δ_1_ = 0.2. The vicinity of *μ* = Δ_2_ is shown in the insert: the solid (red) *T*/Δ_1_ = 5 × 10^−3^, dashed (green) *T*/Δ_1_ = 1.5 × 10^−2^, dash-dotted (blue) *T*/Δ_1_ = 3 × 10^−2^.

Once the carrier imbalance function, *n*(*T*, *μ*, Δ_1_, Δ_2_, …, Δ_*N*_), is found, the entropy per electron can be calculated using Eqs. (1) and (4). In Fig. 1(a) and (b) we show the dependence *s*(*μ*) for graphene and silicene, respectively, for three different values of *T*. Since the entropy per electron is an odd function of *μ*, only the region *μ* > 0 is shown. In the case of silicene we express *μ* and *T* in the units of a smaller gap, Δ_1_. The dependence *s*(*μ*) in the vicinity of the second gap, *μ* = Δ_2_ = 2Δ_1_ is shown in the insert of Fig. 1(b) to resolve the spike structure for three temperatures lower than the values on the main plot.

The most prominent feature that we find in Fig. 1(a) and (b) is a sharp peak observed for the chemical potential in the temperature vicinity of the Dirac point, $|\mu | \sim T$.

If the chemical potential is inside the gap but it is not very close to the Dirac point, $T\ll |\mu | < {\rm{\Delta }}$, and $T\ll {\rm{\Delta }}-|\mu |$, the entropy per particle in a gapped graphene is

10 $$s(T,\mu ,{\rm{\Delta }})\simeq {\rm{s}}{\rm{i}}{\rm{g}}{\rm{n}}(\mu )[\frac{{\rm{\Delta }}-|\mu |}{T}+1+\frac{T}{{\rm{\Delta }}+T}].$$

Near the Dirac point, $|\mu |\ll T\ll {\rm{\Delta }}$, one finds

11 $$s(T,\mu ,{\rm{\Delta }})\simeq \frac{\mu {\rm{\Delta }}}{{T}^{2}}[1+O({e}^{-{\rm{\Delta }}/T})].$$

If the chemical potential crosses the Dirac point at *T* = 0, the transition from hole-like to electron-like carriers is singular. Eqs. (10) and (11) show how the temperature smears it. The peak inside the gap is mainly due to the specific dependence of the chemical potential on the electron density. Indeed, since *s* = ∂*S*(*T*, *μ*)/∂*n* = (∂*S*(*T*, *μ*)/∂*μ*)(∂*μ*/∂*n*), the dependence *s*(*μ*) is governed by the sharpest function in the product. The chemical potential grows rapidly at the small density *n* and then quickly reaches the value $|\mu |\simeq {\rm{\Delta }}$, where the derivative ∂*μ*/∂*n* becomes small. The peaked behavior of *s* may be considered as a smoking gun for the gap opening in gapped Dirac materials.

Near the Lifshitz transition points: *μ* = ±Δ, we observe that the dependences *s*(*μ*) are monotonic functions, so that these points are not marked by spikes. This is typical for any system where DOS has just one discontinuity^2^. Nevertheless, the entropy per particle quantization rule for graphene *s*(*μ* = ±Δ) = ±2 ln2 is fulfilled. One can see that in both panels of Fig. 1, at low temperatures all curves cross each other near this point. The corresponding value *s* = 2 ln2 is shown by the dotted line. This numerical result can be confirmed analytically. For $T\ll {\rm{\Delta }}$ we obtain

12 $$s(T,\mu ={\rm{\Delta }},{\rm{\Delta }})=2\,\mathrm{ln}\,2+\frac{{\pi }^{2}-12\,{\mathrm{ln}}^{2}2}{3}\frac{T}{{\rm{\Delta }}}+O\,({T}^{2})\mathrm{.}$$

Now we briefly discuss the effect of broadening of the energy levels due to the scattering from static defects. Let us smear the DOS function (2) by convoluting it with the Lorentzian, *γ*/[*π*(*ω* ^2^ + *γ* ^2^)], where *γ* is the scattering rate. In the regime $\gamma \ll T\ll {\rm{\Delta }}$ one finds

13 $$s(T,\mu ={\rm{\Delta }},{\rm{\Delta }})=2\,{\rm{l}}{\rm{n}}\,2\,[1-\frac{\gamma }{T}(\frac{1}{\pi {\rm{l}}{\rm{n}}2}+\frac{T}{{\rm{\Delta }}})].$$

Eq. (13) shows that the universality of the low temperature entropy per particle is broken by the disorder if the mean free path becomes comparable with the thermal diffusion length.

The case Δ = 0 deserves a special attention. In this limit, Eq. (9) acquires a simple form (see the Methods, Eqs (24) and (25)). For the entropy per particle one finds

14 $$s(T,\mu ,0)=\{\begin{array}{c}\frac{\mu }{T}(1-\frac{{\mu }^{2}}{{T}^{2}}\frac{1}{6\,{\rm{l}}{\rm{n}}\,2}),\quad |\mu |\ll T,\\ \frac{{\pi }^{2}}{3}\frac{T}{\mu },\quad T\ll |\mu |.\end{array}$$

It is important to note that the second line of Eq. (14) if multiplied by the factor *k*_*B*_/*e* yields the Seebeck coefficient for a free electron gas^15^. Moreover, the general expression for *s* = −∂*μ*/∂*T*, Eq. (25) reproduces the thermal power *S* that can be extracted from the results based on the Kubo formalism^16^ that validates the thermodynamic approach of ref. 17.

The presence of the second gap in silicene and similar materials, Δ_2_ > Δ_1_, results in the appearance of the peak in *s*(*μ*) ≈ ±2ln2/3 near the point *μ* = ±Δ_2_, as seen in Fig. 1(b). The corresponding value *s* = 2ln2/3 is shown by the dotted line. This peak can be considered as a signature of the second Lifshitz transition which occurs if *μ* crosses Δ_2_. Indeed, as it was shown for the quasi-2DEG in ref. 2 the peak structure in *s*(*μ*) develops only if the number of discontinuities in the DOS, *N* ≥ 2. Thus, these perspective Dirac materials, where the spin orbit interaction plays a very important role allow the simplest realization of the *N* = 2 case with two discontinuities on both electron and hole sides of the total DOS.

**Figure 2:**
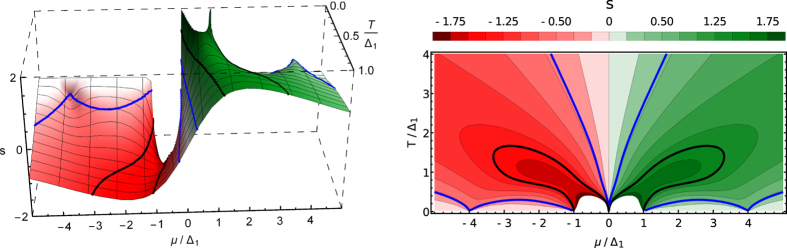
The entropy per electron *s* as functions of the chemical potential *μ* and temperature *T* in the units of Δ_1_. The gap Δ_2_ = 4Δ_1_. Left panel: 3D plot. Right panel: Contour plot.

Figure 2 shows the 3D and density plots of *s* as a function of *μ*/Δ_1_ and *T*/Δ_1_. To be specific, we assumed that Δ_1_ is the smallest of the gaps and chose Δ_2_ = 4Δ_1_. The black and blue lines correspond to the contours of constant values *s* = ±2ln2 and *s* = ±2ln2/3, respectively. The range of *s* in the 3D plot is restricted by −2 ≤ *s* ≤ 2, so that only the peaks at *μ* = ±Δ_2_ can be seen.

A more careful examination of Fig. 1(b) shows that the peak occurring near *μ* = Δ_2_ is somewhat shifted to smaller than Δ_2_ values of *μ*. Looking at Fig. 2(b) and its insert one can trace how the position of this peak moves towards the point (*μ* = Δ_2_, *T* = 0) as the temperature decreases. In Fig. 2(a) the increase of its height can be seen. Close to this point ($T\ll {{\rm{\Delta }}}_{2}$) we obtain analytically

15 $$s(T,\mu =\pm {{\rm{\Delta }}}_{2})=\pm [\frac{2\,{\rm{l}}{\rm{n}}\,2}{3}+\frac{{\pi }^{2}-4\,{{\rm{l}}{\rm{n}}}^{2}2}{9}\frac{T}{{{\rm{\Delta }}}_{2}}].$$

In what concerns the behaviour the silicene’s entropy per particle close to the smallest gap, Δ_1_, it is described by Eq. (12) with Δ replaced by Δ_1_.

**Figure 3:**
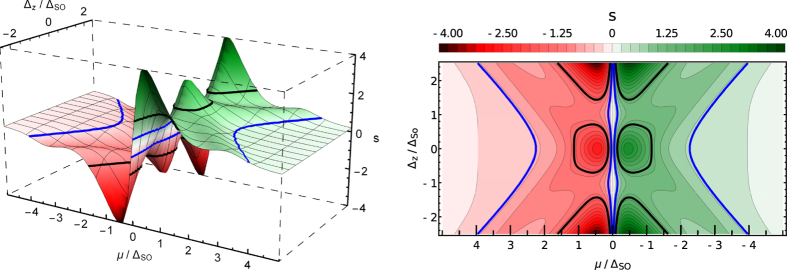
The entropy per electron *s* as functions of the chemical potential *μ* and Δ_*z*_ in the units of Δ_SO_. The temperature *T* = 0.3Δ_SO_. Left panel: 3D plot. Right panel: Contour plot.

Recent successes in fabrication of silicene field-effect transistors^18^ offers the opportunity of a direct measurement of the entropy per particle in silicene. In the prospective experiment, a double gate structure would be needed that enables one to tune *μ* and Δ_*z*_ independently. Such a situation is modelled in Fig. 3, where we show the 3D and density plots of *s* as a function of *μ*/Δ_SO_ and Δ_*z*_/Δ_SO_. As in Fig. 2, the black and blue lines correspond to the contours of constant values *s* = ±2ln2 and *s* = ±2ln2/3, respectively. The points Δ_*z*_ = ±Δ_SO_ correspond to the case where Δ_1_ = 0 and Δ_2_ = 2Δ_SO_ or Δ_1_ = 2Δ_SO_ and Δ_2_ = 0, so that the system experiences a transition from two to one gap spectrum. For |Δ_*z*_| < Δ_SO_ the system is a topological insulator and for |Δ_*z*_| > Δ_SO_ it is a band insulator.

## Discussion

We presented original analytical expressions for the entropy per particle in a wide energy range for various Dirac materials. Basing on them we have predicted the characteristic spikes of the entropy per particle at the Lifshitz topological transition points in several 2D Dirac systems. The magnitude of spikes is quantized at low temperatures and is independent of material parameters. The quantized spikes are expected to occur in silicene and germanene. They can also be found in the gapped graphene in the presence of Zeeman splitting and in quasi two-dimensional Dirac and Weyl materials. Note that the same quantization of entropy and spikes occur in a 2DEG in the presence of Zeeman splitting^1^, see the Methods.

Our results are based on the assumption that the function *f*(*ε*) in the DOS (2) is continuous. Although this assumption is quite general, it is not fulfilled, for example, in a bilayer graphene. The overall behavior of the entropy per electron ∂*S*/∂*n* as a function of the electronic chemical potential may be used as a tool for characterization of the electronic dispersion in novel crystal structures. The crucial point is that ∂*S*/∂*n* is related to the temperature derivative ∂*μ*/∂*T* via the thermodynamic Maxwell relation (1). The last value, as was mentioned in Introduction, can be directly measured using the experimental approach developed in ref. 1. It appears that this technique has a three orders of magnitude higher resolution than the other methods and thus it can be very helpful in probing interaction effects in 2D electron systems. The measurements of the entropy per particle can also be used to study the effect of interactions on the DOS in graphene, because the renormalization of the Fermi velocity due to electron-electron interactions^19^ modifies the function *s*(*n*).

## Methods

### Relationship between the carrier density and carrier imbalance

At thermal equilibrium, the total density of electrons in a nonrelativistic system can be expressed as

16 $${n}_{{\rm{t}}{\rm{o}}{\rm{t}}}(T,\mu )={\int }_{-{\rm{\infty }}}^{{\rm{\infty }}}d\varepsilon D(\varepsilon ){f}_{FD}(\frac{\varepsilon -\mu }{T}),$$

where *f*_*FD*_(*ε*) = 1/[exp(*ε*/*T*) + 1] is the Fermi-Dirac distribution function and we set *k*_*B*_ = 1. In a relativistic theory, for example, in QED the number of electrons or positrons is not conserved, while a conserving number operator is needed to build the statistical density matrix^20^. In QED, the conserved quantity if the difference of the numbers of positively and negatively charged particles: electrons and positrons.

In the Dirac materials the “relativistic” nature of carriers is encoded in the symmetric DOS function, *D*(*ε*) = *D*(−*ε*). Accordingly, it is convenient to operate with the difference between the densities of electrons and holes instead of the total density of electrons^21, 22^. The difference is given by

17 $$n(T,\mu )={\int }_{-\infty }^{\infty }d\varepsilon D(\varepsilon )[{f}_{FD}(\varepsilon -\mu )\theta (\varepsilon )-[1-{f}_{FD}(\varepsilon -\mu )]\theta (-\varepsilon )]=-\frac{1}{2}{\int }_{-\infty }^{\infty }d\varepsilon D(\varepsilon )\tanh \,\frac{\varepsilon -\mu }{2T}.$$

The last equation can be rewritten in the form of Eq. (3). One can verify that the carrier imbalance *n*(*T*, *μ*) and the total carrier density *n*_tot_(*T*, *μ*) are related by the expression *n*(*T*, *μ*) = *n*_tot_(*T*, *μ*) − *n*_hf_, where *n*_hf_ is the density of particles for a half-filled band (in the lower Dirac cone) ${n}_{{\rm{hf}}}={\int }_{-\infty }^{\infty }d\varepsilon D(\varepsilon )\theta (-\varepsilon )$. Consequently, there is no difference whether the entropy per particle in Eq. (1) is defined via the total carrier density *n*_tot_ or the carrier imbalance *n*.

### General expressions for ∂*n*/∂*T* and ∂*n*/∂*μ*

The first temperature derivative in Eq. (4) depends on whether the chemical potential *μ* hits the discontinuity of the DOS *D*(*ε*) given by Eq. (2). Differentiating Eq. (3) over the temperature one obtains

18 $$\frac{{\rm{\partial }}n(T,\mu )}{{\rm{\partial }}T}=\frac{{\rm{s}}{\rm{i}}{\rm{g}}{\rm{n}}(\mu )}{4T}{\int }_{-{\rm{\infty }}}^{{\rm{\infty }}}d\varepsilon D(\varepsilon )[\frac{\varepsilon -|\mu |}{2T}\frac{1}{{\cosh }^{2}\frac{\varepsilon -|\mu |}{2T}}-\frac{\varepsilon +|\mu |}{2T}\frac{1}{{\cosh }^{2}\frac{\varepsilon +|\mu |}{2T}}].$$

Changing the variable *ε* = 2*Tx* ± |*μ*| in two terms and changing the limits of integration, one obtains

19 $$\frac{\partial n(T,\mu )}{\partial T}={\rm{sign}}(\mu ){\int }_{0}^{\infty }dx[D(|\mu |+2Tx)-D(|\mu |-2Tx)]\frac{x}{{\cosh }^{2}x}\mathrm{.}$$

If the DOS *D*(*ε*) has a continuous derivative at the point *ε* = |*μ*|, where ${{\rm{\Delta }}}_{i} < |\mu | < {{\rm{\Delta }}}_{i+1}$, one can expand $D(|\mu |+2Tx)-D(|\mu |-2Tx)\simeq 4TxD^{\prime} (|\mu |)$. Then integrating over *x* we arrive at Eq. (5)

20 $$\frac{\partial n(T,\mu )}{\partial T}\simeq 4T{\rm{sign}}(\mu )D^{\prime} (|\mu |){\int }_{0}^{\infty }\frac{{x}^{2}dx}{{\cosh }^{2}x}={\rm{sign}}(\mu )D^{\prime} (|\mu |)\frac{{\pi }^{2}}{3}T\mathrm{.}$$

On the other hand, at the discontinuity points *μ* = ±Δ_*J*_ at *T* → 0, we arrive at Eq. (6).

The second derivative in Eq. (4) in the zero temperature limit is just the DOS. Indeed, we have

21 $$\frac{{\rm{\partial }}n(T,\mu )}{{\rm{\partial }}\mu }=\frac{1}{8T}{\int }_{-{\rm{\infty }}}^{{\rm{\infty }}}d\varepsilon D(\varepsilon )[\frac{1}{{\cosh }^{2}\frac{\varepsilon +\mu }{2T}}+\frac{1}{{\cosh }^{2}\frac{\varepsilon -\mu }{2T}}]=D(\mu ),\,\,T\to 0.$$

This is because $(1/4T){\cosh }^{-2}(x/2T)\to \delta (x)$ for *x* → 0. Substituting the DOS given by Eq. (2) to Eq. (21) we arrive at Eq. (7).

### Explicit expressions for the derivatives ∂*n*/∂*T* and ∂*n*/∂*μ* for the Dirac materials

The carrier imbalance for a gapped graphene is given by Eq. (9). The corresponding derivatives are

22 $${(\frac{{\rm{\partial }}n}{{\rm{\partial }}\mu })}_{T}=\frac{2}{\pi {\hslash }^{2}{v}_{F}^{2}}[\frac{{\rm{\Delta }}}{2}(\tanh \,\frac{\mu -{\rm{\Delta }}}{2T}-\,\tanh \,\frac{\mu +{\rm{\Delta }}}{2T})+T({\rm{l}}{\rm{n}}(2\,\cosh \,\frac{\mu -{\rm{\Delta }}}{2T})+\,{\rm{l}}{\rm{n}}(2\,\cosh \,\frac{\mu +{\rm{\Delta }}}{2T}))]$$

and

23 $$\begin{array}{ccc}{(\frac{{\rm{\partial }}n}{{\rm{\partial }}T})}_{\mu } & = & \frac{2}{\pi {\hslash }^{2}{v}_{F}^{2}}[2{\rm{\Delta }}\,{\rm{l}}{\rm{n}}\,\frac{1+\exp (\frac{\mu -{\rm{\Delta }}}{T})}{1+\exp (-\frac{\mu +{\rm{\Delta }}}{T})}+2T{{\rm{L}}{\rm{i}}}_{2}(-{e}^{-\frac{\mu +{\rm{\Delta }}}{T}})-2T{{\rm{L}}{\rm{i}}}_{2}(-{e}^{\frac{\mu -{\rm{\Delta }}}{T}})\\  &  & -\mu \,{\rm{l}}{\rm{n}}(2\,\cosh \,\frac{\mu -{\rm{\Delta }}}{2T})-\mu \,{\rm{l}}{\rm{n}}(2\,\cosh \,\frac{\mu +{\rm{\Delta }}}{2T})+\frac{{\rm{\Delta }}}{T}\frac{\mu \,\sinh ({\rm{\Delta }}/T)+{\rm{\Delta }}\,\sinh \,\mu /T}{\cosh \,{\rm{\Delta }}/T+\,\cosh \,\mu /T}].\end{array}$$

Eqs (10)–(12) and (15) are obtained using the low-temperature expansions of the derivatives, Eqs (22) and (23).

### Dirac materials with Δ = 0

If Δ = 0 Eq. (9) reduces to

24 $$n(T,\mu )=\frac{2{T}^{2}}{\pi {\hslash }^{2}{v}_{F}^{2}}[{{\rm{L}}{\rm{i}}}_{2}(-{e}^{-\frac{\mu }{T}})-{{\rm{L}}{\rm{i}}}_{2}(-{e}^{\frac{\mu }{T}})].$$

Using Eq. (4) we obtain the general expression

25 $${(\frac{{\rm{\partial }}\mu }{{\rm{\partial }}T})}_{n}=\frac{\mu }{T}-\frac{1}{{\rm{l}}{\rm{n}}(2\,\cosh \,\frac{\mu }{2T})}[{{\rm{L}}{\rm{i}}}_{2}(-{e}^{-\frac{\mu }{T}})-{{\rm{L}}{\rm{i}}}_{2}(-{e}^{\frac{\mu }{T}})].$$

### Quantization of entropy in the presence of Zeeman splitting

In the 2DEG in the presence of Zeeman splitting considered in the Supplementary material of ref. 1 the carrier density reads

26 $$n(\mu ,T)=\frac{m}{4\pi }T[{\rm{l}}{\rm{n}}(1+{e}^{(\mu +Z)/T})+\,{\rm{l}}{\rm{n}}(1+{e}^{(\mu -Z)/T})].$$

Here *Z* is the Zeeman splitting energy and *m* is the carrier mass. One can show that the entropy per particle in this case also obeys the quantization rule

27 $${\frac{{\rm{\partial }}S}{{\rm{\partial }}n}|}_{\mu =-Z}=2\,{\rm{l}}{\rm{n}}\,2,\quad {\frac{{\rm{\partial }}S}{{\rm{\partial }}n}|}_{\mu =Z}=\frac{2\,{\rm{l}}{\rm{n}}\,2}{3},\quad T\to 0.$$
